# Proteome-Wide Mendelian Randomization and Colocalization Analysis Identify Therapeutic Targets for Knee and Hip Osteoarthritis

**DOI:** 10.3390/biom14030355

**Published:** 2024-03-15

**Authors:** Mingrui Zou, Zhenxing Shao

**Affiliations:** 1 Department of Sports Medicine, Peking University Third Hospital, Institute of Sports Medicine of Peking University, Beijing 100191, China; 2110301145@stu.pku.edu.cn; 2 Beijing Key Laboratory of Sports Injuries, Engineering Research Center of Sports Trauma Treatment Technology and Devices, Ministry of Education, Beijing 100191, China

**Keywords:** GWAS, mendelian randomization, osteoarthritis, plasma proteins, therapeutic targets

## Abstract

Osteoarthritis (OA) is a common degenerative disease. Although some biomarkers and drug targets of OA have been discovered and employed, limitations and challenges still exist in the targeted therapy of OA. Mendelian randomization (MR) analysis has been regarded as a reliable analytic method to identify effective therapeutic targets. Thus, we aimed to identify novel therapeutic targets for OA and investigate their potential side effects based on MR analysis. In this study, two-sample MR, colocalization analysis, summary-data-based Mendelian randomization (SMR) and Mendelian randomization phenome-wide association study (MR-PheWAS) were conducted. We firstly analyzed data from 4907 plasma proteins to identify potential therapeutic targets associated with OA. In addition, blood expression quantitative trait loci (eQTLs) data sources were used to perform additional validation. A protein–protein interaction (PPI) network was also constructed to delve into the interactions among identified proteins. Then, MR-PheWASs were utilized to assess the potential side effects of core therapeutic targets. After MR analysis and FDR correction, we identified twelve proteins as potential therapeutic targets for knee OA or hip OA. Colocalization analysis and additional validation supported our findings, and PPI networks revealed the interactions among identified proteins. Finally, we identified MAPK3 (OR = 0.855, 95% CI: 0.791–0.923, *p* = 6.88 × 10^−5^) and GZMK (OR = 1.278, 95% CI: 1.131–1.444, *p* = 8.58 × 10^−5^) as the core therapeutic targets for knee OA, and ITIH1 (OR = 0.847, 95% CI: 0.784–0.915, *p* = 2.44 × 10^−5^) for hip OA. A further MR phenome-wide association study revealed the potential side effects of treatments targeting MAPK3, GZMK, and ITIH1. This comprehensive study indicates twelve plasma proteins with potential roles in knee and hip OA as therapeutic targets. This advancement holds promise for the progression of OA drug development, and paves the way for more efficacious treatments of OA.

## 1. Introduction

Osteoarthritis (OA) is the most common degenerative disorder of the joint, which typically involves lesions in articular cartilage, subchondral bone, ligaments, capsule, and synovial membrane [1]. According to the statistics from the epidemiology and register center in Lund university, in 2012, the proportion of OA patients in the Skåne region of southern Sweden was 26.6%. It is estimated that by 2032, the proportion will increase from 26.6% to 29.5% for OA, from 13.8% to 15.7% for knee OA, and from 5.8 to 6.9% for hip OA [2,3]. In recent years, the number of OA patients has been continuously increasing, bringing significant economic burden to society. In high-income countries, the medical expenses for OA account for 1~2.5% of the gross domestic product [3]. Among all OA, knee and hip OA are the most common, posing a threat to the health of millions of people [4]. Therefore, finding effective diagnostic and therapeutic strategies for OA is urgent, with knee and hip OA being the focus of work.

However, OA treatment remains challenging. Firstly, the drugs recommended by international guidelines for treating OA can only alleviate symptoms and cannot cure OA or effectively delay its progression [5]. Furthermore, the safety of these drugs remains to be debated. For example, paracetamol can relieve pain, but it increases the risk of cardiovascular and renal adverse events [6]. In addition, corticosteroids are effective for OA patients, but they are harmful to articular cartilage [7]. In the past few decades, drugs targeting specific molecules for OA have been developed, such as TNF inhibitor (adalimumab) [8], IL-1 inhibitor (anakinra) [9], and IκB kinase inhibitor (SAR113945) [10]. However, in clinical trials, there is little evidence to support that these drugs effectively alleviate pain and improve function in OA patients [11]. Discovering new drug targets for OA and developing effective targeted drugs remain big challenges with great potential.

Plasma proteins are significant components of the blood and perform pivotal functions in the circulation [12]. In addition, as diseases progress, the concentration and activity of plasma proteins are often dysregulated, making them therapeutic targets for various diseases [13]. At present, some studies have focused on the development of plasma protein biomarkers and drug targets for OA, but there are still significant limitations [14,15]. Firstly, traditional clinical observational studies are susceptible to various confounding factors, leading to inaccurate results. In addition, traditional research cannot determine whether there is a causal relationship between plasma proteins and OA, as well as the directionality of the causal relationship. In addition, plasma proteins may have the same expression trend in various diseases, so the specificity of biomarkers cannot be guaranteed. Therefore, it is still challenging to obtain biomarkers and drug targets for OA only through observational research and we need research strategies that are more robust and reliable.

Mendelian randomization (MR) analysis is a reliable analytic method to evaluate the causal association between exposures and outcomes, which has been widely used in discovering novel therapeutic targets and drug repurposing [16,17]. The MR analysis is based on three core assumptions: (1) Correlation assumption: The genetic instrumental variables (IVs) are strongly correlated to the exposure. (2) Independence assumption: The IVs are not affected by confounding factors related to both the exposure and outcome. (3) Exclusivity assumption: The IVs are only allowed to exert effect on the outcome via the exposure [18]. Genome-wide association studies (GWASs) of gene expression levels and protein levels could help identify expression quantitative trait loci (eQTLs) and protein quantitative trait loci (pQTLs) [19,20]. By integrating eQTLs, pQTLs and summary data from disease GWASs, genes, or proteins with causal effects on diseases can be identified as potential therapeutic targets through MR analysis. Compared to traditional observational clinical studies, Mendelian randomization analysis can effectively minimize the effect of confounding factors and reverse causality, thus providing definitive causal relationships [21]. However, there have been only a limited number of studies conducted integrating disease GWASs, eQTLs and pQTLs, especially for knee and hip OA.

**Figure 1:**
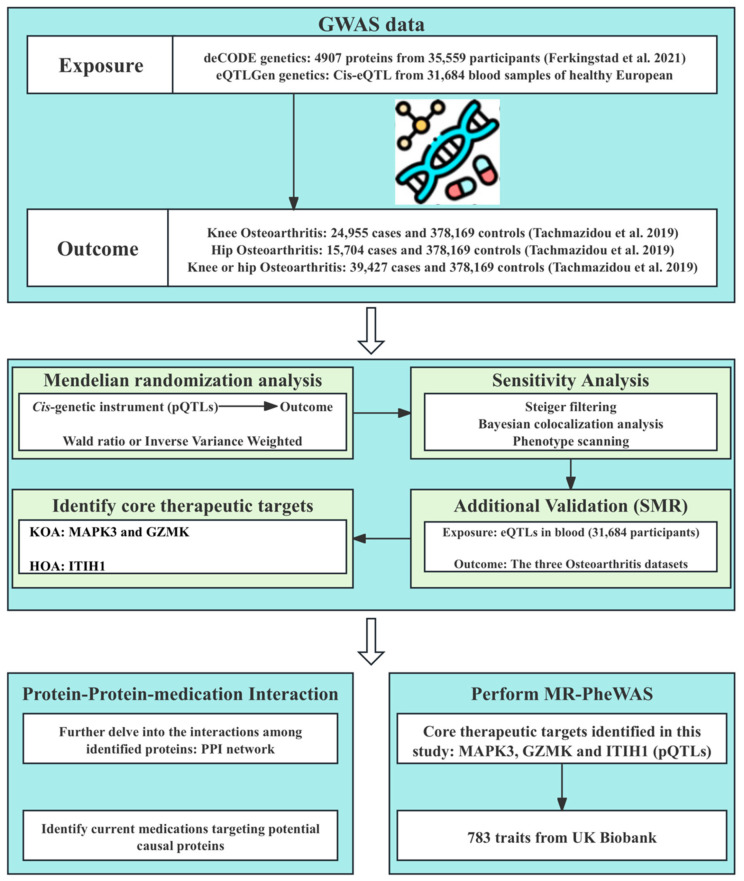
Flowchart of the MR study identifying therapeutic targets for knee and hip OA. GWAS, genome-wide association study; pQTLs, protein quantitative trait loci; eQTLs, expression quantitative trait loci; SMR, summary-data-based Mendelian randomization; KOA, knee osteoarthritis; HOA, hip osteoarthritis; PPI, Protein–protein interaction; MR-PheWAS, Mendelian randomization phenome-wide association study [20,22].

In this study, we performed a systematic proteome-wide MR analysis to identify novel therapeutic targets for knee and hip OA. Firstly, we performed a two-sample MR analysis to examine the causal effects of plasma proteins on OA using pQTLs data from a large-scale integration study [20] and GWAS summary data of OA [22]. In addition, Steiger filtering analysis, Bayesian colocalization analysis, and Phenotype scanning were conducted to verify the robustness of our results. Furthermore, we performed summary-data-based Mendelian randomization (SMR) analysis using blood eQTLs data to validate our findings from the perspective of gene expression. In addition, we constructed a protein–protein interaction (PPI) network to depict the interactions among identified proteins, and we sourced information of existing OA therapeutics and drugs targeting identified proteins. Finally, we evaluated the potential side effects of the identified targets for OA treatment using MR phenome-wide association studies. The overall study design is illustrated in Figure 1. Further details of materials and methods are provided as follows.

## 2. Materials and Methods

Data used in this study are all from existing publications or public databases, which had acquired ethical approval and informed consent, and no extra ethical approval was required.

### 2.1. Data Sources

Plasma pQTLs data were obtained from a large-scale integration study by Ferkingstad et al. [20], who provided pQTLs of 4907 plasma proteins from 35,559 Icelanders. To obtain *cis*-pQTLs, we selected pQTLs according to the following criteria: (1) The pQTLs presented genome-wide significant association (*p* < 5 × 10^−8^). (2) The pQTLs were located outside the major histocompatibility complex (MHC) region (chr6, 29–33 Mb). (3) The assumption of independence was satisfied (linkage disequilibrium (LD) clumping $r^{2}$ < 0.001). (4) pQTLs were *cis*-acting (pQTLs within a 1000 kb window across the corresponding protein-coding sequences). (5) The pQTLs were not weak IVs (with F-statistics greater than 10). (6) Palindromic single nucleotide polymorphisms (SNPs) and SNPs containing missing data were eliminated. Finally, 6238 cis-acting SNPs for 1729 proteins were included (Supplementary Table S1).

The summary statistics of knee and hip OA were obtained from a large GWAS. The dataset of knee OA included 403,124 individuals (24,955 cases and 378,169 controls), the dataset of hip OA included 393,873 individuals (15,704 cases and 378,169 controls) and the dataset of knee or hip OA included 417,596 individuals (39,427 cases and 378,169 controls). The OA cases were obtained from UK Biobank and Arthritis Research UK Osteoarthritis Genetics (arcOGEN). The OA cases were identified based on clinical evidence or radiographic evidence (Kellgren–Lawrence grade ≥ 2). The controls were obtained from United Kingdom Household Longitudinal Study (UKHLS), which is a longitudinal panel survey of 40,000 UK households (from England, Scotland, Wales, and Northern Ireland) [22].

For additional validation, the blood eQTL dataset was obtained from eQTLGen (https://www.eqtlgen.org/, accessed on 1 January 2024). The dataset contains *cis*-eQTLs of 16,987 genes from 31,684 blood samples from healthy European ancestry [23]. For the identified 12 plasma proteins (CRYZ, MAPK3, LGALS3, GZMK, MAX, CFHR3, OMG, DNAJB12, ITIH1, ULK3, USP8, and CSK), we selected common (minor allele frequency (MAF) > 0.01) eQTLs SNPs significantly (*p* < 5 × 10^−8^) associated the expression of corresponding genes in blood. Similarly to primary MR analysis, only strong (with F-statistics greater than 10) *cis*-eQTLs (eQTLs within a 1000 kb window across the encoded gene) were included as IVs. Finally, there were no eligible eQTLs in blood for CFHR3 and ITIH1, and we conducted additional validation for the remaining 10 proteins.

All the information of summary statistics datasets used in this study are listed in Supplementary Table S2.

### 2.2. MR and SMR Analysis

#### 2.2.1. MR Analysis

In the primary MR analysis, plasma proteins were used as exposures, and OA as outcomes. When there was only one pQTL as IV for the plasma protein, Wald ratio (WR) method was used to evaluate the causal effect [24]. If two or more IVs were available, inverse-variance weighted (IVW) method was used [25]. Statistical results are presented in the form of odds ratios (OR) and 95% confidence intervals (95% CI) with a nominal significant threshold of *p*-value < 0.05. To avoid false positive results, false discovery rate (FDR) correction was applied. Statistically significant resulted was defined as FDR < 0.05 [26]. If a protein presented nominally significant association but did not after correction, we considered it a suggestively significant result. Proteins that presented significant association with OA will be further investigated by SMR analysis and MR phenome-wide association study, etc. The primary MR analysis was performed using “TwoSampleMR” R package (V.0.5.8) in the R software package (V.4.3.1).

To make our findings robust and reliable, Cochran’s Q test and MR Egger intercept test were utilized to assess potential heterogeneity and horizontal pleiotropy after IVW method was applied (*p* > 0.05 indicates the absence of heterogeneity or horizontal pleiotropy) [27,28]. In addition, for IVW analysis, fixed effect model and random effect model were used. When MR analysis did not detect significant heterogeneity, fixed effect model was used; conversely, random effect model was used.

#### 2.2.2. SMR Analysis

For additional validation, we conducted summary-data-based Mendelian randomization (SMR) analysis to generate effect estimates. By using summary-level data from GWAS and eQTL studies, we evaluate the causal association between the expression levels of genes corresponding to the identified protein targets and OA [19]. In addition, we used SMR software package (V.1.03) to perform allele harmonization and analysis [19]. In additional validation, the *p*-value threshold of SMR analysis was 0.05.

In addition, we conducted heterogeneity in dependent instruments (HEIDI) test to assess whether the association was caused by linkage scenario. *p*-value of HEIDI test < 0.05 indicates that the observed association was presumably due to linkage disequilibrium. The HEIDI test was also performed with the help of SMR software (V.1.03) [19].

### 2.3. Bayesian Colocalization Analysis and Steiger Filtering Analysis

We performed Bayesian colocalization analysis to find out whether the causal effects of proteins on OA were driven by linkage disequilibrium or each genomic locus contained a single variant affecting both the protein and OA [29]. In our study, we employed “coloc” R package (V.5.2.3) with the following settings: (1) $P_{1}=1\times 10^{-4}$: The prior probability of the SNP was associated only with trait 1. (2) $P_{2}=1\times 10^{-4}$: The probability of the SNP was associated only with trait 2. (3) $P_{12}=1\times 10^{-5}$: The probability of the SNP was associated with both traits. The Bayesian colocalization analysis evaluated the support for 5 hypotheses: (1) H0: no association with the two traits; (2) H1: association only with trait 1; (3) H2: association only with trait 2; (4) H3: association with both traits, but they have distinct causal variants; (5) H4: association with both traits, and they share the same causal variant. Each hypothesis has a posterior probability (PP). If the posterior probability of hypothesis 4 (PPH4) is ≥0.8, the two traits are considered to have strong support of colocalization [30]. In this study, we employed pQTL data of proteins and eQTL data of corresponding genes to perform colocalization analysis with OA GWAS data, respectively.

By using “TwoSampleMR” R package (V.0.5.8), Steiger filtering analysis was performed to assess whether our results were affected by reverse causality. If the direction is “TRUE” and the *p*-value < 0.05, reverse causality is considered absent [29].

### 2.4. Phenotype Scanning

With the help of the “phenoscanner” tool [31], we performed phenotype scanning to find out whether the identified pQTLs were strongly associated with other traits and had pleiotropic effects. If the pQTLs met the following criteria, they were regarded to have pleiotropic effects: (1) pQTLs had observed association which reached genome-wide significance (*p* < 5 × 10^−8^); (2) pQTLs presented associations with clearly known risk factors of OA.

### 2.5. Protein–Protein Interaction (PPI) Network and Drug Identification

To investigate the interactions among identified proteins, we constructed protein–protein interaction (PPI) networks (proteins with *p*-value of IVW or Wald ratio < 0.05 in primary MR analysis were included). The Search Tool for the Retrieval of Interacting Genes (STRING, V.12.0, https://string-db.org/, accessed on 13 January 2024) was used to perform PPI analyses, with the minimum required interaction score at 0.7 (high confidence) [32]. In addition, we obtained target information for existing knee OA and hip OA therapeutics and collected the information of drugs targeting the identified proteins from the DrugBank database (V.5.0, https://go.drugbank.com, accessed on 16 January 2024) [33]. This existing knowledge will help us understand the targeted therapy of OA in depth and promote therapeutic drug development.

### 2.6. Classification Hierarchy of Proteins as Potential Drug Targets

After performing MR analysis, FDR correction, additional validation, and colocalization analysis, we divided the 12 identified proteins into four categories (tiers) based on the following criteria: (1) the protein passes additional validation (*p*-value of SMR analysis < 0.05); (2) HEIDI test is passed (*p*-value > 0.05); (3) pQTLs of the protein demonstrate colocalization with OA; (4) eQTLs of the protein demonstrate colocalization with OA; (5) The direction of effects in primary MR analysis and SMR analysis are consistent (both ORs are greater than 1 or less than 1). Under the prerequisite of meeting the principle of directionality (criteria (5)), proteins that pass 5 criteria are tier 1 targets, proteins that pass 4 criteria are tier 2 targets, proteins that pass 3 criteria are tier 3 targets, and the remaining proteins are tier 4 targets. Two proteins without additional validation are considered separately. Proteins that meet criteria (3) are tier 2 targets, or they are tier 4 targets.

### 2.7. MR Phenome-Wide Association Study

To further explore the side effects of the three core proteins (MAPK3, GZMK, and ITIH1) associated with knee or hip OA, we performed MR phenome-wide association studies. The *cis*-pQTLs of three core proteins were used as exposures, and summary statistics of diseases in the UK Biobank cohort with a sample size up to 408,961 individuals were used as outcomes [34]. In the process of conducting GWASs, Scalable and Accurate Implementation of Generalized Mixed Model (SAIGE V.0.29) method was used to adjust for imbalanced case–control ratios by researchers [34]. To ensure sufficient statistical power, we selected 783 traits (diseases) with more than 500 cases from the 1403 traits (diseases) as outcomes. These 783 traits or diseases were defined according to the International Classification of Diseases and Related Health Problems (ICD-9/-10) [35]. The detailed information of the 783 traits or diseases were presented in Supplementary Table S3. We performed MR analysis via the IVW method, and causal association was considered statistically significant if corrected *p*-value < 0.05 [25]. In addition, Cochran’s Q test and MR Egger intercept test were utilized to evaluate the heterogeneity and horizontal pleiotropy (*p* > 0.05 indicates the absence of heterogeneity or horizontal pleiotropy) [27,28].

## 3. Results

### 3.1. MR Results

**Figure 2:**
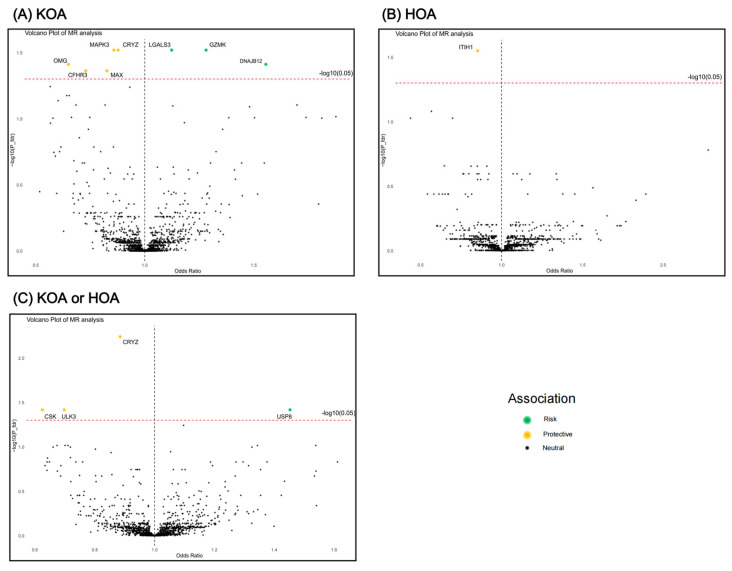
Volcano plots of MR analysis. The causal association between plasma proteins and risk of (**A**) KOA, (**B**) HOA, and (**C**) KOA or HOA. KOA, knee osteoarthritis; HOA, hip osteoarthritis.

**Figure 3:**
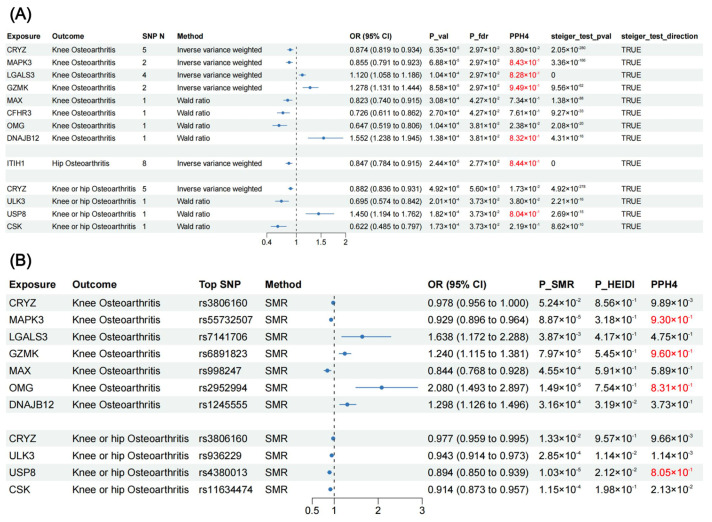
MR and SMR analysis results in the discovery and validation phase. (**A**) Forest plot for MR results between plasma pQTLs and OA. (**B**) Forest plot for SMR results between blood eQTLs and OA. SNP N, number of single nucleotide polymorphism; OR, odds ratio; CI, confidence interval; P_val, *p* value; P_fdr, *p* value corrected by FDR method; PPH4, posterior probability of hypothesis 4; SMR, summary-data-based Mendelian randomization; P_SMR, *p* value of SMR analysis; P_HEIDI, *p* value of heterogeneity in dependent instruments test; pQTLs, protein quantitative trait loci; OA, osteoarthritis; eQTLs, expression quantitative trait loci.

After FDR correction, MR analysis revealed twelve proteins that presented significant association with OA (Figure 2 and Figure 3), including CRYZ, MAPK3, LGALS3, GZMK, MAX, CFHR3, OMG, DNAJB12, ITIH1, ULK3, USP8, and CSK. For knee OA, three proteins could increase its risk: LGALS3 (OR = 1.120, 95% CI: 1.058–1.186, *p* = 1.04 × 10^−4^), GZMK (OR = 1.278, 95% CI: 1.131–1.444, *p* = 8.58 × 10^−5^), and DNAJB12 (OR = 1.552, 95% CI: 1.238–1.945, *p* = 1.38 × 10^−4^); and five proteins could decrease its risk: CRYZ (OR = 0.874, 95% CI: 0.819–0.934, *p* = 6.35 × 10^−5^), MAPK3 (OR = 0.855, 95% CI: 0.791–0.923, *p* = 6.88 × 10^−5^), MAX (OR = 0.823, 95% CI: 0.740–0.915, *p* = 3.08 × 10^−4^), CFHR3 (OR = 0.726, 95% CI: 0.611–0.862, *p* = 2.70 × 10^−4^), and OMG (OR = 0.647, 95% CI: 0.519–0.806, *p* = 1.04 × 10^−4^). ITIH1 (OR = 0.847, 95% CI: 0.784–0.915, *p* = 2.44 × 10^−5^) decreased the risk of hip OA. For the knee or hip OA dataset, four proteins demonstrated significant association: CRYZ (OR = 0.882, 95% CI: 0.836–0.931, *p* = 4.92 × 10^−6^), ULK3 (OR = 0.695, 95% CI: 0.574–0.842, *p* = 2.01 × 10^−4^), USP8 (OR = 1.450, 95% CI: 1.194–1.762, *p* = 1.82 × 10^−4^), and CSK (OR = 0.622, 95% CI: 0.485–0.797, *p* = 1.73 × 10^−4^). Steiger filtering ensured the directionality of the causal association (Figure 3), and heterogeneity and horizontal pleiotropy were not detected for the twelve proteins in the primary MR analysis (Supplementary Table S4).

### 3.2. SMR Results (Additional Validation)

In the additional validation phase, we performed summary-data-based Mendelian randomization (SMR) analysis using blood eQTLs data to validate our findings from the perspective of gene expression (Figure 3). Expression levels of MAPK3 (OR = 0.929, 95% CI: 0.896–0.964, *p* = 8.87 × 10^−4^) and MAX (OR = 0.844, 95% CI: 0.768–0.928, *p* = 4.55 × 10^−4^) reduced the risk of knee OA. Expression levels of LGALS3 (OR = 1.638, 95% CI: 1.172–2.288, *p* = 3.87 × 10^−3^), GZMK (OR = 1.240, 95% CI: 1.115–1.381, *p* = 7.97 × 10^−5^), OMG (OR = 2.080, 95% CI: 1.493–2.897, *p* = 1.49 × 10^−5^), and DNAJB12 (OR = 1.298, 95% CI: 1.126–1.496, *p* = 3.16 × 10^−5^) increased the risk of knee OA. Expression levels of CRYZ (OR = 0.977, 95% CI: 0.959–0.995, *p* = 1.33 × 10^−2^), ULK3 (OR = 0.943, 95% CI: 0.914–0.973, *p* = 2.85 × 10^−4^), USP8 (OR = 0.894, 95% CI: 0.850–0.939, *p* = 1.03 × 10^−5^), and CSK (OR = 0.914, 95% CI: 0.873–0.957, *p* = 1.15 × 10^−4^) could reduce the risk of knee or hip OA. Except for DNAJB12, ULK3, and USP8, the HEIDI test indicates that the association between the expression levels of other seven proteins and OA were not caused by linkage disequilibrium (*p*-value > 0.05).

Interestingly, the effects of gene expression levels of OMG and USP8 on OA were opposite to the effects of expression levels of corresponding plasma proteins on OA, which worth further research.

### 3.3. Colocalization Analysis and Phenotype Scanning for Causal Proteins

In primary MR analysis, Bayesian colocalization analysis strongly suggested that MAPK3 (PPH4 = 0.843), LGALS3 (PPH4 = 0.828), GZMK (PPH4 = 0.949), DNAJB12 (PPH4 = 0.832), ITIH1 (PPH4 = 0.844), and USP8 (PPH4 = 0.804) shared the same variant with OA (Figure 3, Supplementary Table S5). In additional validation phase, MAPK3 (PPH4 = 0.930), GZMK (PPH4 = 0.960), OMG (PPH4 = 0.831), and USP8 (PPH4 = 0.805) demonstrated colocalization with OA (Figure 3, Supplementary Table S5).

After phenotype scanning, ULK3 and USP were found to be associated with hypertension, monocyte percentage of white cells and other vascular or heart problems. MAX demonstrated association with red blood cell traits and height. In addition, we observed CFHR3 to be associated with macular degeneration, and OMG and ITIH1 to be associated with height. MAPK3 showed an association with blood cell traits, fat mass, fat-free mass, water mass, impedance, height, and basal metabolic rate. LGALS3 displayed association with mean platelet volume. However, there was no evidence to suggest direct association between these phenotypes and OA (Supplementary Table S6).

Guided by MR analysis, additional validation and colocalization analysis, the 12 identified proteins were divided into four categories. For knee OA, MAPK3 and GZMK were tier 1 targets, and LGALS3 was a tier 2 target. MAX and DNAJB12 were tier 3 targets, and CRYZ, CFHR3 and OMG were tier 4 targets. For hip OA, ITIH1 was a tier 2 target. CRYZ and CSK were tier 3 targets, and ULK3 and USP8 were tier 4 targets for knee or hip OA. (Supplementary Table S7).

### 3.4. Interactions among MR-Prioritized Proteins and Their Association with Current Medication

**Figure 4:**
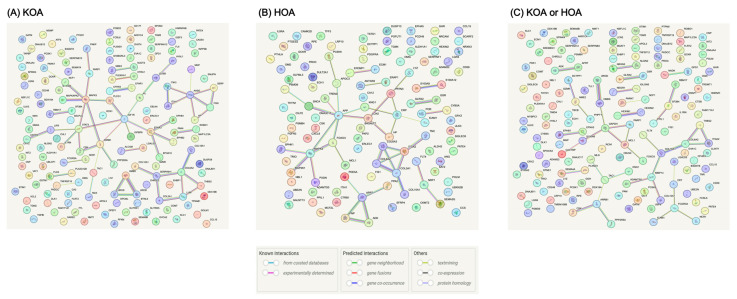
Protein–protein interaction (PPI) network among identified protein targets and suggestive protein targets. (**A**) KOA, (**B**) HOA, and (**C**) KOA or HOA. KOA, knee osteoarthritis; HOA, hip osteoarthritis.

In the primary MR analysis, we identified 150 proteins that at least presented suggestively significant association with knee OA, 112 with hip OA, and 148 with knee or hip OA. The PPI network revealed the interactions among these proteins (Figure 4). Proteins that presented suggestively significant association with OA and interacted with 12 identified causal proteins might provide new insights for us to further investigate the association between identified protein targets and OA. For example, MAPK3 interacted with multiple proteins including PLCG1, PAPR1, NCF1, MAPKAPK2, STAT3, and ARRB1, and MAPK3 was located at the core position of the PPI network. In addition, LGALS3 interacted with ALCAM, CFHR3 interacted with CFHR1, OMG interacted with TNFRSF19 and CSK interacted with ARRB1 (Figure 4).

With the help of the DrugBank database, we summarized 8 existing medications for knee and hip OA with their corresponding targets, and 23 medications targeting the identified proteins. The detailed information is presented in Supplementary Tables S8 and S9. Despite the known association, there are still many identified OA protein targets, and no drugs interacting with them have been found yet. So, there is still great potential for the development of targeted drugs for OA in the future.

### 3.5. MR Phenome-Wide Association Studies of Core Therapeutic Targets of OA

**Figure 5:**
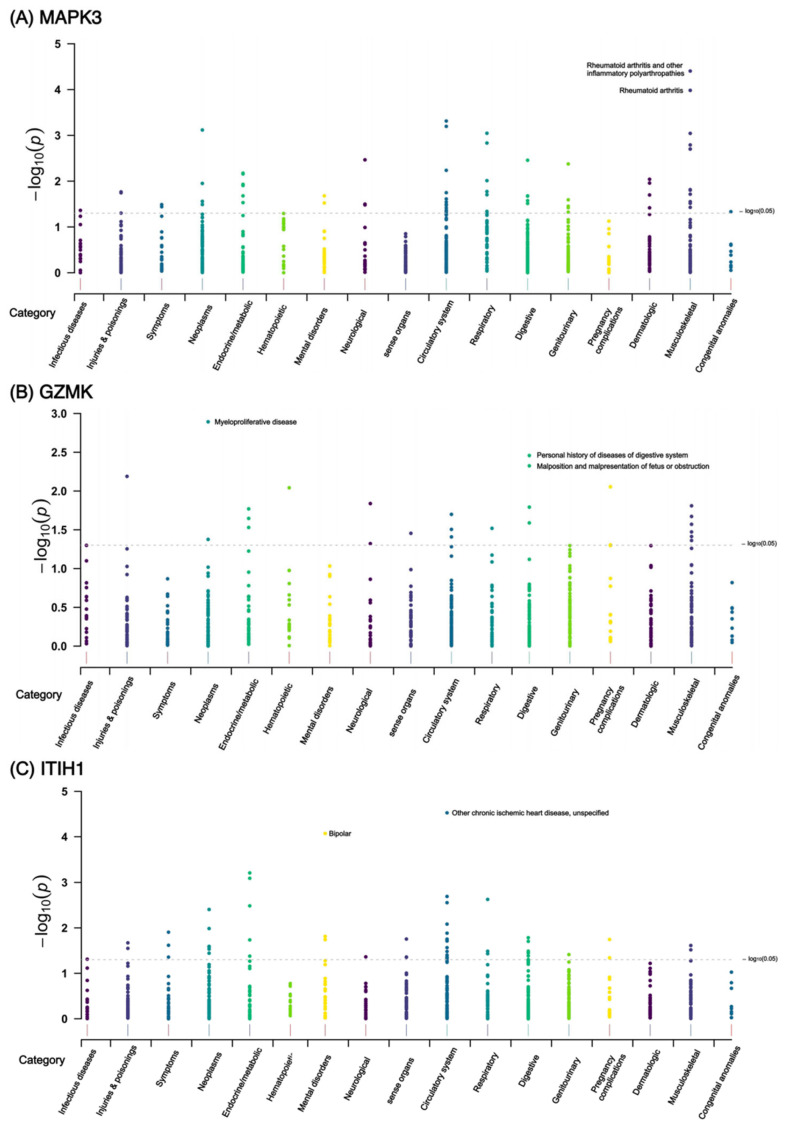
Manhattan plot for MR phenome-wide association study of (**A**) MAPK3, (**B**) GZMK, and (**C**) ITIH1. A dot represents a disease or trait.

**Figure 6:**
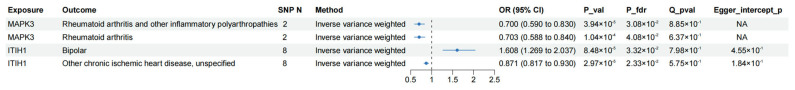
Forest plot for significant results in MR phenome-wide association study.

MR phenome-wide association studies were performed to investigate whether side effects existed in treatments targeting MAPK3, GZMK, and ITIH1 (the most important identified targets for knee and hip OA). A total of 783 traits or diseases in the UK Biobank were utilized for MR screening, and the Manhattan plots for the results are presented in Figure 5. Using IVW method, four significant associations were identified (Figure 6). We discovered that higher plasma MAPK3 levels could reduce the risk of rheumatoid arthritis (RA), while higher plasma ITIH1 levels might increase the risk of bipolar and reduce the risk of chronic ischemic heart disease (Figure 6). Cochran’s Q test and MR Egger intercept test failed to find heterogeneity and horizontal pleiotropy. We did not find significant causal association between GZMK and other diseases, and it was mainly associated with digestive system diseases and musculoskeletal system diseases (Figure 5). The summary results are presented in Supplementary Tables S10–S12.

## 4. Discussion

To our knowledge, this is the first study to combine MR analysis, SMR analysis, Bayesian colocalization analysis and MR phenome-wide association study to investigate therapeutic targets for knee and hip OA. Primary MR analysis identified eight causal proteins (CRYZ, MAPK3, LGALS3, GZMK, MAX, CFHR3, OMG, and DNAJB12) for knee OA, one causal protein (ITIH1) for hip OA, and four causal proteins (CRYZ, ULK3, USP8, and CSK) for knee or hip OA. SMR analysis, the HEIDI test, and colocalization analysis further support our findings. Finally, we identified MAPK3 and GZMK as the most important targets for knee OA (Tier 1 targets), and ITIH1 for hip OA (Tier 2 target). MR phenome-wide association study emphasized the potential beneficial indications and side effects of treatments targeting MAPK3, GZMK, and ITIH1, which could help us understand these three genes from a more comprehensive perspective.

In this study, we employed an integrative analysis which combined MR and SMR with colocalization analysis to find novel drug targets for knee and hip OA. As we know, causality assessed by MR analysis might be interfered by reverse causality [35]. Therefore, we performed Steiger filtering analysis to ensure the directionality of causal effects. In addition, to minimize the bias caused by horizontal pleiotropy, we selected *cis*-pQTLs as IVs, and conducted Bayesian colocalization analysis and phenotype scanning. With 0.8 as the threshold for PPH4, we further identified proteins that share the same variant with OA and further eliminate the bias [36]. Phenotype scanning revealed that eight of the twelve identified proteins (ULK3, USP8, MAX, CFHR3, OMG, ITIH1, MAPK3, and LGALS3) were associated with other traits, but none of the traits could fully explain the causal association between identified proteins and OA. However, it should be noted that MAPK3 is associated with fat mass and basal metabolic rate. As we know, knee and hip joint are weight-bearing joints, and excessive obesity may increase the risk of osteoarthritis by increasing joint load [37]. Although fat mass and basal metabolic rate cannot be equated with obesity, bias in the causal association between MAPK3 and knee OA should still be considered. However, with the support of additional validation and colocalization analysis, we still consider MAPK3 as a reliable drug target for knee OA, but its role should be carefully interpreted. In MR phenome-wide association studies, we found that plasma MAPK3 levels could reduce the risk of RA, while higher plasma ITIH1 levels might increase the risk of bipolar and reduce the risk of chronic ischemic heart disease. A comprehensive understanding of the side effects of these identified proteins will help to maximize their therapeutic potential and provide new insights for targeted therapy of OA. Based on all the outcomes, we divided the twelve proteins into four categories to prioritize their therapeutic potential (Supplementary Table S7). Tier 1 and tier 2 targets (MAPK3, LGALS3, GZMK, ITIH1) should be paid more attention to, being appealing candidates for targeted therapy of OA.

Mitogen-activated protein kinase 3 (MAPK3), also known as extracellular signal-regulated kinase 1 (ERK1), is a member of MAP kinase family, which regulates various cellular processes such as cell proliferation, cell differentiation and cell cycle progression [38]. Our study identified MAPK3 (ERK1) as a protective tier 1 target for knee OA. Consistently with our study, one study found that ERK1 deficient chondrocytes downregulated the expression of NRF2 and upregulated the expression of BACH1, and the lack of ERK1 can promote cartilage degradation and accelerate the progression of OA [39]. In addition, Ansari et al. reported that ERK1/2 plays an important role in mitochondrial fission and apoptosis of chondrocytes induced by Dynamin-related protein 1 [40]. At present, various drugs targeting MAPK3 have been developed, among which sulindac and acetylsalicylic acid have been applied in the treatment of OA [41,42]. However, there are still many limitations. Firstly, the drugs targeting MAPK3 for OA are mainly NSAID drugs, which can only alleviate pain but cannot effectively delay the progression of OA. Secondly, our MR phenome-wide association study indicates that MAPK3 can reduce the risk of RA without any other side effects. However, MAPK3 is not the only target of existing drugs, and it is unknown whether there are any side effects when the drugs act on other targets. Therefore, there is still a substantial journey ahead in achieving precise treatment of OA targeting MAPK3.

Galectin-3 (LGALS3) is unique in the family of galectins, and it is distributed in multiple sites of the body, including the intestines, skeleton, brain, and kidneys. Its main functions are associated with angiogenesis, inflammatory response, and tumor progression [43]. Consistently with our findings, previous studies have reported that the expression level of LGALS3 is higher in the synovium of OA and RA patients [44]. In addition, animal experiments have also demonstrated that LGALS3 could induce joint swelling and OA-like lesions [45]. To be specific, LGALS3 could induce the secretion of pro-inflammatory cytokines such as IL-6, TNF-*α*, and amplify the inflammatory response [46]. In addition, LGALS3 could promote the degradation of cartilage and inhibit the differentiation of osteoblasts, thereby inhibiting joint remodeling and exacerbating lesions [47,48]. In our study, LGALS3 was identified as a tier 2 target for knee OA, the plasma level of which might increase the risk of knee OA. However, at present, there are very few drugs targeting LGALS3, and the only few drugs are in the experimental and investigation stage. Therefore, LGALS3 has great potential in targeted therapy of knee OA and warrants further exploration in future research.

Granzyme K (GZMK) is a proapoptotic serine protease secreted by granules, which plays both intercellular and extracellular roles [49]. At present, there are few studies reporting the role of GZMK in OA or the association between GZMK and OA, and we did not identify any drugs targeting GZMK. However, the granzyme family has been found to be associated with RA. GZMK has been found to be highly expressed in RA patients, promoting the degradation of extracellular matrix and the release of inflammatory cytokines. In our study, as a tier 1 target, GZMK may increase the risk of knee OA, and we anticipate the development of drugs targeting GZMK for OA treatment in the future.

Inter-alpha-trypsin inhibitor heavy chain 1 (ITIH1) is a member of inter-alpha-trypsin inhibitor family, which has been implicated in multiple inflammatory diseases [50,51]. Interestingly, there is some inconsistency between our study and previous studies. Several studies have proven that ITIH1 could serve as a biomarker for knee OA, and high levels of ITIH1 are associated with the early occurrence of knee OA [52,53]. However, our study identified ITIH1 as a protective target for hip OA, and ITIH1 presented suggestively protective association with knee OA. This contradiction might be caused by confounding factors and reverse causality, which cannot be avoid in traditional studies. However, more studies are needed to find out the correct association between ITIH1 and OA. In addition, MR phenome-wide association study revealed that ITIH1 might increase the risk of bipolar, which should also be emphasized in drug development.

There are eight proteins identified as tier 3 or tier 4 targets of OA in our study (Supplementary Table S7). We believe that these proteins have the potential to become drug targets of OA, but there are certain limitations. Given the limited research on the role of these proteins in OA, further research is imperative to validate their dependability. Currently, there are some drugs targeting these proteins. For example, Dicoumarol, Cannabidiol, and Nabiximols are drugs targeting CRYZ, and Fostamatinib is an inhibitor of ULK3 [54,55,56]. However, these drugs are only applicable to other diseases such as deep vein thrombosis and multiple sclerosis, and there is no evidence to suggest that they are beneficial for OA patients.

This study has some strengths. Firstly, the F-statistics of IVs were all greater than > 10, indicating the absence of weak instrument bias [57]. In addition, *cis*-pQTLs are closer to their corresponding genes, which can effectively minimize horizontal pleiotropy when used as IVs. Thirdly, multiple analysis strategies were used in this study to make our results robust and reliable. In addition to primary MR analysis, we conducted SMR analysis and Bayesian colocalization analysis to further validate our findings. In addition, we performed MR phenome-wide association study to investigate the side effects of identified targets. Fourthly, we created PPI networks to present the interactions among proteins and summarized existing knowledge on therapeutics for OA to complement our findings. Finally, while conducting MR analysis, we also conducted Steiger filtering analysis to demonstrate the directionality of causal relationships and conducted phenotype scanning to exclude the interfere of confounding factors. Therefore, compared to traditional observational studies, our study can provide accurate estimates of causal effects.

However, several limitations cannot be ignored. Firstly, we only selected *cis*-pQTLs as IVs, and most proteins had no more than three SNPs, which limit the application of some MR analysis strategies such as Cochran’s Q test, MR Egger intercept test and MR pleiotropy residual sum and outlier (MR-PRESSO) test. Secondly, the high-quality GWAS data associated with OA are limited. Therefore, our analysis is limited to knee OA and hip OA. In the future, with high-quality GWAS data of OA in other sites of the body being available, potential drugs targeting OA in other sites will be identified. In addition, all the data used in our study are based on European populations, so whether our findings are equally applicable to other racial or ethnic groups remains to be debated. Data of non-European ancestry are required to further validate our findings. In addition, our study was limited to exploring the causal association between plasma proteins and OA. However, it is important to note that OA involves lesions in multiple tissues, including cartilage, synovium, and bone. Unfortunately, due to the scarcity of relevant eQTLs and pQTLs data, our study is unable to delve deeper into the tissue-level analysis. Finally, we did not conduct cell and animal experiments to explore the mechanisms of causal association between these proteins and OA. Future research can be based on our research findings to conduct basic experiments and explore the specific mechanisms of OA targeted therapy.

## 5. Conclusions

In conclusion, we identified twelve plasma proteins that presented causal association with knee or hip OA through MR analysis. With the help of SMR and colocalization analysis, we divided these twelve proteins into four categories. The top-tier proteins (Tier 1 and 2) are the most promising candidates for targeted therapy of OA (MAPK3, LGALS3, and GZMK for knee OA, and ITIH1 for hip OA). However, further basic studies are needed to validate these findings and provide new insights into targeted therapy for knee and hip OA.

## Data Availability

The sources of the GWAS summary statistics utilized in this study are available in Supplementary Table S2.

## Supplementary Materials

The following supporting information can be downloaded at: https://www.mdpi.com/article/10.3390/biom14030355/s1, Table S1. Instrumental variables of plasma proteins used in MR analysis; Table S2. The sources for all statistical summary datasets used in this study; Table S3: The information of 783 diseases incorporated in the MR-PheWAS analysis; Table S4. The results of Cochran’s Q test and MR Egger intercept test in primary MR analysis; Table S5. Colocalization results of pQTLs for 12 proteins and eQTLs for 10 genes with OA-associated SNPs; Table S6. Investigating the Previous Genome-Wide Significant Associations of SNPs as Genetic Instruments for Potential Causal Proteins; Table S7. Distinct target groups of identified proteins; Table S8. Current medications and corresponding targets for knee and hip OA treatment; Table S9. Current medications targeting identified potential causal proteins; Table S10: MR-PheWAS results of Plasma MAPK3 in IVW method; Table S11: MR-PheWAS results of Plasma GZMK in IVW method; Table S12: MR-PheWAS results of Plasma ITIH1 in IVW method.
